# Early innovations in opioid use disorder treatment and harm reduction during the COVID-19 pandemic: a scoping review

**DOI:** 10.1186/s13722-021-00275-1

**Published:** 2021-11-13

**Authors:** Noa Krawczyk, Adetayo Fawole, Jenny Yang, Babak Tofighi

**Affiliations:** 1 Center for Opioid Epidemiology and Policy, Department of Population Health, NYU Grossman School of Medicine, 180 Madison Ave, Room 4-12, New York, NY USA; 2 New York University, School of Global Public Health, New York, NY USA; 3 NYU Grossman School of Medicine, New York, NY USA; 4 Department of Population Health, NYU Grossman School of Medicine, New York, NY USA

**Keywords:** Opioids, Opioid use disorder, Treatment, Harm reduction, COVID-19, Telehealth, Low-threshold, Emergency

## Abstract

**Background:**

The COVID-19 pandemic has exerted a significant toll on the lives of people who use opioids (PWUOs). At the same time, more flexible regulations around provision of opioid use disorder (OUD) services have led to new opportunities for facilitating access to services for PWUOs. In the current scoping review, we describe new services and service modifications implemented by treatment and harm reduction programs serving PWUO, and discuss implications for policy and practice.

**Methods:**

Literature searches were conducted within PubMed, LitCovid, Embase, and PsycInfo for English-language studies published in 2020 that describe a particular program, service, or intervention aimed at facilitating access to OUD treatment and/or harm reduction services during the COVID-19 pandemic. Abstracts were independently screened by two reviewers. Relevant studies were reviewed in full and those that met inclusion criteria underwent final data extraction and synthesis (n = 25). We used a narrative synthesis approach to identify major themes around key service modifications and innovations implemented across programs serving PWUO.

**Results:**

Reviewed OUD treatment and harm reduction services spanned five continents and a range of settings from substance use treatment to street outreach programs. Innovative service modifications to adapt to COVID-19 circumstances primarily involved expanded use of telehealth services (e.g., telemedicine visits for buprenorphine, virtual individual or group therapy sessions, provision of donated or publicly available phones), increased take-home medication allowances for methadone and buprenorphine, expanded uptake of long-acting opioid medications (e.g. extended-release buprenorphine and naltrexone), home delivery of services (e.g. MOUD, naloxone and urine drug screening), outreach and makeshift services for delivering MOUD and naloxone, and provision of a safe supply of opioids.

**Conclusions:**

The COVID-19 pandemic has posed multiple challenges for PWUOs, while simultaneously accelerating innovations in policies, care models, and technologies to lower thresholds for life-saving treatment and harm reduction services. Such innovations highlight novel patient-centered and feasible approaches to mitigating OUD related harms. Further studies are needed to assess the long-term impact of these approaches and inform policies that improve access to care for PWUOs.

## Introduction

The coronavirus disease 2019 (COVID-19) pandemic has exerted a significant toll on the lives of people who use opioids (PWUOs). Rising overdose deaths since the COVID-19 pandemic have been reported in the U.S. [1], Canada [2, 3] and Europe [4]. High rates of overdose during the COVID-19 pandemic have been attributed to numerous risk factors, including increased isolation and despair, unpredictable changes to routes and contents of the drug supply, and decreased access to treatment and harm reduction services [5–8]. In addition to overdose harms, PWUOs often experience a high prevalence of medical comorbidities, housing instability, criminal justice involvement, stigma, and reduced access to health services, exacerbating risks for COVID-19 related morbidity and mortality [9, 10].

Given these circumstances, easing access to life-saving opioid use disorder (OUD) services has become more urgent than ever. Medications for opioid use disorder (MOUD), including methadone, buprenorphine, and extended-release naltrexone, are highly effective at reducing overdose risk [11, 12] and improving health outcomes among PWUOs [13]. Naloxone administration plays a critical role in reversing opioid overdoses when made widely available to both first responders and laypeople [14]. Other harm reduction services, including syringe services programs, overdose prevention sites, and drug checking services, are critical for reducing transmission of infectious diseases, providing overdose prevention education and supplies, and delivering support to vulnerable PWUO [15]. Despite their efficacy, strict regulations around OUD treatment, insufficient investment of resources in harm reduction, and stigma against PWUOs have historically limited the public health impact of these programs [16, 17].

Access to OUD services has become especially precarious during the COVID-19 pandemic, as service closures, lower staff availability, and concerns around COVID-19 transmission have led to reduced service availability or utilization at a time when these services are needed most [18, 19]. At the same time, relaxed regulations and emergency mandates around OUD treatment and harm reduction services introduced during the pandemic have brought new hope for the possibility of expanding lower threshold care options during the pandemic [17]. In the U.S., for example, concerns about COVID-19 transmission led the Substance Abuse and Mental Health Services Administration (SAMHSA) and Drug Enforcement Administration (DEA) to relax treatment regulations around provision of MOUD. This included expanding limits on take-home doses of methadone of up to 14 days for moderately stable and up to 28 days for highly stable patients. In addition, the office of Health and Human Services (HHS), along with the Attorney General, waived the U.S. Ryan Haight Act's in-person examination requirement for controlled substances, permitting the initiation of buprenorphine treatment entirely via telemedicine for the duration of the COVID-19 emergency [20]. In British Columbia, the government announced interim Risk Mitigation Guidance during the COVID-19 crisis, which permitted prescribing of opioid medication alternatives to illicit opioids, such as hydromorphone and morphine, to help manage withdrawal symptoms among PWUO. Also known as ‘Safe supply,’ this risk mitigation strategy was designed to provide a safer alternative to an increasingly toxic drug supply and reduce overdose risk while enabling self-isolation among PWUO [21, 22]. In Australia, prescribers and dispensers have been encouraged to allow for longer duration of MOUD prescriptions and take-home medications to minimize travel and in-person contact during the COVID-19 pandemic [23]. These changes that were implemented as a result of seemingly temporary pandemic circumstances therefore offer an unprecedented opportunity for practitioners, policymakers and researchers to expand low-barrier services and assess their potential utility beyond the COVID-19 emergency.

In the current study, we conducted a scoping review of articles published in 2020 to understand how OUD treatment and harm reduction programs globally have innovated and adapted their services to COVID-19 circumstances and identify ongoing gaps in delivering services for PWUOs. To this end, the objective of this review was to: (1) identify characteristics of programs that adapted OUD services during the pandemic; (2) describe innovative services or service modifications that emerged as a result of COVID-19 circumstances; and (3) discuss implications for policy and practice responses to meet the needs of PWUO during and beyond the current COVID-19 pandemic.

## Methods

We conducted this review in accordance with the Preferred Reporting Items of Systematic Reviews and Meta-Analyses for Scoping Review (PRISMA-ScR) checklist [24] (Appendix 1: Table 3).

### Eligibility criteria

We sought peer-reviewed English language articles published in 2020 that described either new programs or adaptations of existing programs to deliver evidence-based treatment with MOUD (methadone, buprenorphine, or extended-release naltrexone) or other overdose prevention and harm reduction services for PWUO under COVID-19 emergency conditions. We limited our search to articles that described particular programs/interventions and not those that offered aggregate data on service modifications from multiple providers or programs.

### Search strategy

We consulted with a librarian at the New York University Health Sciences Library to develop the search approach and extraction process. We adopted a broad search strategy to identify peer-reviewed literature from multiple electronic databases in November 2020. A second search was conducted in February 2021 to include articles published between November and December of 2020. Electronic database searches included PubMed (initial: 265 results, second search: 97 results), Embase (initial: 344, second search: 119), PsycInfo (initial: 32, second search: 17), and LitCovid, a curated literature hub that tracks up-to-date scientific information about the 2019 novel Coronavirus in PubMed [25, 26] (initial: 182, second search: 104).

The search term strategy combined keywords related to opioids, treatment, naloxone, harm reduction and COVID-19 using the following keywords: (opioid* OR opiate* OR Heroin OR suboxone OR subutex OR buprenorphine OR methadone OR naltrexone OR vivitrol OR naloxone OR Narcan OR "prescription abuse" OR "opiate addiction" OR "opiate overdose" OR "people who use drugs" OR "Harm reduction" OR "syringe exchange" OR "needle exchange" OR "safe injection") AND (COVID* OR COVID-19 OR sars-cov-2 OR coronavirus OR pandemic OR quarantine).

### Screening and selection

Initial database search results were imported into Endnote, and subsequently uploaded into Covidence, a subscription-based systematic review tool [27]. Upon importing references to Covidence, duplicate search results were automatically removed and processed for Title/ Abstract screening. Two reviewers (A.F. & J.Y.) independently screened titles and abstracts with full blinding for initial eligibility. Disagreements were resolved through discussions with the first and senior authors (N.K. & B.T.). Articles found to meet eligibility underwent full-text review by at least two members of the team and content was extracted from eligible articles. Full study team consensus was sought to select the final list of articles that met inclusion criteria. This process was repeated during each phase of article extraction (November, 2020 and February, 2021).

### Data extraction and synthesis of findings

Program characteristics collected from articles that met inclusion criteria included: author and month of publication; location of program (e.g. state/province, country); care setting (e.g. substance use treatment, primary care, outreach/harm reduction); name of program/managing organization; managing organization type (e.g. academic institution, non-for-profit organization); article study design (e.g. commentary/program description, case report); whether programs offered OUD treatment, harm reduction services (including naloxone), or both; and types of services/medications offered (e.g. methadone, naloxone). We also collected information related to service modifications during the COVID-19 pandemic including: whether the program was newly created or a modification of an existing program prior to the pandemic; unique strategies and services implemented to meet patient needs during the COVID-19 pandemic; any reported outcomes of the program(s); key obstacles and facilitators encountered; and lessons reported for OUD service policies and practices.

We used a narrative synthesis approach [28] to iteratively identify and classify major themes and concepts across articles based on study objectives. N.K. initially conducted the thematic analysis and synthesis, and A.F., J.Y. and B.T. provided analytical input to refine themes and effectively synthesize and report the most important lessons and implications from the reviewed studies.

## Results

**Fig. 1:**
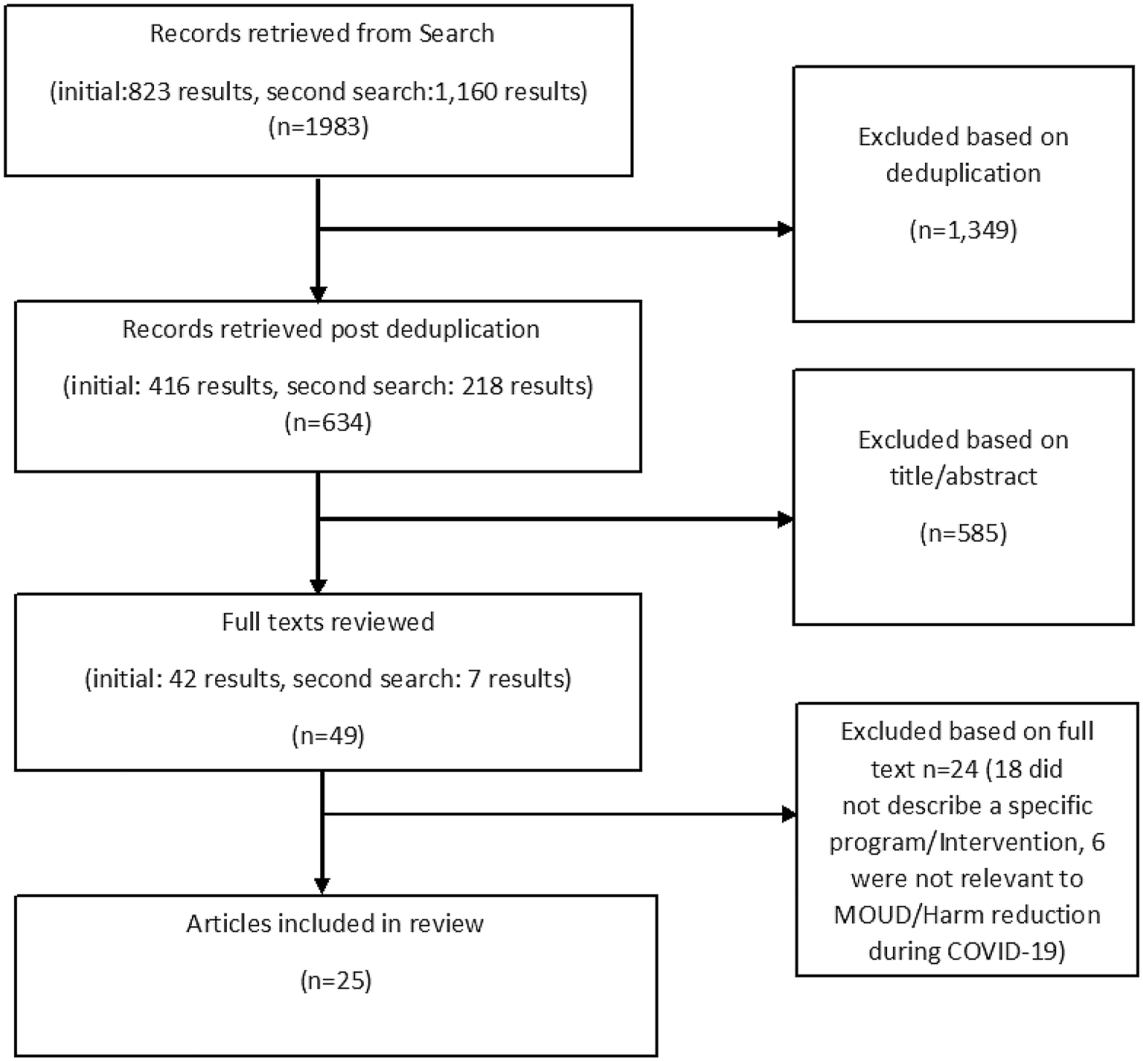
Selection criteria process for reviewed articles

Our search resulted in 634 total articles, out of which 49 went through full-text extraction, and 25 met final inclusion criteria for this review (Fig. 1).

### Program characteristics

Descriptive characteristics of programs in the 25 articles included in the review are summarized in Table 1. The majority of studies described programs in the U.S. (52%), but also spanned countries in Europe, Asia, Oceania, Africa, and other parts of North and Latin America. Studies primarily described adaptations of existing programs (84%) rather than new programs. Programs were primarily affiliated with or directly operated by academic institutions (28%) or independent clinics (28%). The most common care settings described were substance use treatment programs (52%) followed by outreach/harm reduction programs (28%). Most studies discussed OUD treatment services only (84%) or both treatment and harm reduction (e.g. naloxone, syringe distribution; safe supply) (12%), while only one program (4%) discussed harm reduction services alone. Sublingual buprenorphine was the most common medication offered by the described programs (64%), followed by naloxone (48%) and methadone (44%). The majority of study designs were commentaries describing adaptation of OUD services during COVID-19 (72%), while only 16% reported some type of quantitative outcome. This may reflect the timing of the data captured, as articles published in 2020 may not have had sufficient time to gather information on health outcomes.

**Table 1:** Program characteristics of included articles (N = 25)

Article study design/type	N (%)
Commentary or program description	18 (72%)
Research with quantitative outcomes	4 (16%)
Research with qualitative outcomes	2 (8%)
Case report	1 (4%)
Country	
United States	13 (52%)
Puerto Rico	1 (4%)
South Africa	1 (4%)
United Kingdom	1 (4%)
Ireland	1 (4%)
India	2 (8%)
Australia	3 (12%)
Canada	1 (4%)
Spain	1 (4%)
Italy	1 (4%)
Care setting	
Substance use treatment	13 (52%)
Outreach/harm reduction	7 (28%)
Correctional facility	2 (8%)
Mobile clinic	2 (8%)
Primary care/general practitioner	1 (4%)
Type of organization	
Academic institution	7 (28%)
Government/academic partnership	3 (12%)
Government	4 (16%)
Non-for-profit organization	3 (12%)
Independent clinic	7 (28%)
Commercial laboratory	1 (4%)
Services described	
OUD Treatment	21 (84%)
Harm reduction	1 (4%)
OUD Treatment and Harm reduction	3 (12%)
Types of medications offered	
Sublingual buprenorphine	16 (64%)
Extended release buprenorphine	3 (12%)
Methadone	11 (44%)
Extended-release naltrexone	1 (4%)
Naloxone	12 (48%)
New vs. modification of program	
New	4 (16%)
Modification	21 (84%)

### Innovative strategies to adapt services to COVID-19 circumstances

OUD treatment and harm reduction programs employed a range of strategies to adapt their services to the changing circumstances of COVID-19 and ensure services reached people in need. Detailed descriptions of strategies adopted by each program are summarized in Table 2, and are organized by care setting (substance use treatment, primary care, outreach/harm reduction, mobile clinic, and correctional facility). We categorized these strategies into six broad groups described in detail below: expanded telemedicine services; extended take-home medications; uptake of long-acting medications; home delivery of services; outreach and makeshift services; and safe supply of opioids.

**Table 2:** OUD treatment and harm reduction service adaptations in response to COVID-19; Peer-reviewed literature, 2020

Author, month, location	Name of clinic/organization	Care setting	Primary OUD services discussed	Innovative strategies to adapt to COVID-19
Samuel et al., May, 2020 Rhode Island, United States	Brown University and Rhode Island Department of Health	Substance Use Treatment	Buprenorphine	24-h telehealth-based bridge program for buprenorphine initiation and referral to ongoing care
Peavy et al., September, 2020 Washington, USA	Evergreen Treatment Services	Substance Use Treatment	Methadone	Increased take-homes for methadone provided along with smartphone application to allow video-directly observed therapy
Hazan et al., September, 2020 London, United Kingdom	Department of Addiction Psychiatry, Barnet & Haringey Mental Health Trust and Camden & Islington Foundation Trust	Substance Use Treatment	Buprenorphine, Methadone	Extended take-home supplies and lengths of prescriptions; removed supervised consumption and suspended psychosocial services; offered delivery of medications to isolated patients
Straub et al., September, 2020 Victoria, Australia	Long-Acting Injectable Buprenorphine (LAIB) Program St. Vincents Hospital Melbourne	Substance Use Treatment	Buprenorphine	Establishment of rapid access clinic to administer long-acting injectable buprenorphine to reduce travel and crowding
Ghosh, October, 2020 India	National Healthcare System Opioid Agonist Treatment (OAT) Services	Substance Use Treatment	Buprenorphine	Increased number of take-home buprenorphine doses; Increased days and hours of operation; dispensed buprenorphine to family member
Trujols et al., October, 2020 Catelonia, Spain	Methadone Center at Santa Creu i Sant Pau Hospital	Substance Use Treatment	Methadone	Increased number of take-home doses per patient
Basu et al., May, 2020 Chandigarh, India	Drug De-addiction and Treatment Centre, Department of Psychiatry, Postgraduate Institute of Medical Education and Research (PGIMER)	Substance Use Treatment	Buprenorphine, Methadone	Suspended group counseling and urine drug screening; switched psychosocial services to virtual platforms; Increased number of take-home buprenorphine doses prescribed to patients; suspended initiations of new patients;
Vecchio et al., July, 2020 Piedmont, Italy	SUD Specialist Treatment Centre in Biella	Substance Use Treatment	Buprenorphine, Methadone	Suspended or switched psychosocial services to virtual platform; conducted patient assessments and triage via phone/video calls; Increased take home doses of medications for OUD
Quinones et al., November, 2020 Puerto Rico	Corporacion SANOS Clinic, Coalicio´n de Coaliciones Clinic, Clinica Pitirre Iniciativa Comunitaria Clinic	Substance Use Treatment	Buprenorphine, Methadone	Provided psychosocial services via telemedicine; home delivery for medications for OUD; Increased number of days that OUD care was provided to reduce crowding; allowed for telemedicine appointments through patients and providers sitting in separate rooms
Komaromy et al., December, 2020 Massachusetts, USA	The Grayken Center for Addiction at Boston Medical Center-affiliated SUD programs	Substance Use Treatment	Buprenorphine, naloxone, sterile syringe equipment	Offered buprenorphine visits via telehealth; offered harm-reduction interventions for overdose prevention and sterile injection equipment Distributed donated phones to patients who lacked access for telehealth; Extended length of buprenorphine prescriptions; Increased use of extended-release injectable buprenorphine; Created 24-h call-line for addiction treatment; Text messaging or ‘‘on the fly’’ telehealth visits to stay connected with young patients; Addiction consult services switched to telehealth consultations
Hughto et al., October, 2020 Rhode Island, USA	VICTA—Privately owned outpatient substance abuse and mental health treatment program	Substance Use Treatment	Counseling for MOUD patients	Switched counseling of OUD patients to virtual platforms; provided clinicians with laptops to engage in telehealth
McKiever et al., August, 2020 Ohio, USA	Division of Maternal Fetal Medicine, Department of Obstetrics and Gynecology, The Ohio State University College of Medicine	Substance Use Treatment	Psychosocial services for pregnant women receiving OUD treatment	Switched to telehealth for individual and group therapy
Singh and Tikka, October, 2020 Chhattisgarh, India	The Opioid Substitution Clinic at the All India Institute of Medical Sciences (AIIMS), Raipur	Substance Use Treatment	Buprenorphine	Suspended intakes and managed new patients via telehealth; Suspended random urine screening; implemented COVID-19 safety precautions in clinic
Crowley et al., April, 2020 Ireland	The Irish College of General Practitioners and the National Health Service Executive office for Social Inclusion	Primary Care/General Practitioner	Buprenorphine, Methadone	Telephone-based assessment and triage to general practitioner who conducts remote medical management of MOUD following a single in-person visit
Tringale et al., October, 2020 California, United States	Homeless Health Care Los Angeles	Outreach/Harm Reduction Program	Buprenorphine	Telehealth-based buprenorphine treatment through access to sanitized glass telephone booths
Harris et al., May, 2020 Massachusetts, USA	Boston Medical Center-affiliated community drop-in center	Outreach/Harm Reduction Program	Buprenorphine	Telehealth-based buprenorphine initiation through smartphone provided by harm reduction specialist at drop-in clinic
Marcus et al., August, 2020 Tshwane, South Africa	University of Pretoria’s Department of Family Medicine	Outreach/Harm Reduction Program	Methadone	Provided methadone doses in temporary pandemic homeless shelter; Moving stable patients from daily observed treatment to weekly take home dosing
MacKinnon et al., October, 2020 British Columbia, Canada	Medical services within supportive housing units in Vancouver, affiliated with University of British Columbia	Outreach/Harm Reduction Program	Safe supply of opioids, Supervised injection, Methadone, Buprenorphine	Provided pharmaceutical grade alternative to toxic illicit drug supply termed ‘pandemic withdrawal management’; modified supervised injection spaces; Longer MOUD prescriptions; Increasing the number of take-home MOUD doses; switched to virtual platforms for clinical encounters
Castillo et al., November, 2020 Florida, USA	TeleMOUD, University of Miami Miller School of Medicine	Outreach/Harm Reduction Program	Buprenorphine	Telehealth-based buprenorphine treatment initiation advertised via a syringe exchange program and online platform
Courser et al., December, 2020 Ohio, USA	Community Based Take Home Naloxone Programs	Outreach/Harm Reduction Program	Naloxone	Provided individual and group naloxone trainings via telephone and video conferences; promoted naloxone via social media, offered drive through events for naloxone education and distribution, sent mail-order naloxone kits
Nordeck et al., November, 2020 Maryland, USA	Behavioral Health Leadership Institute	Outreach/Harm Reduction Program	Buprenorphine, Naloxone	Telehealth for buprenorphine initiation and maintenance, daily distribution of naloxone kits via a drop-off basket
Wenzel and Fishman, September, 2020 Maryland, USA	Youth Opioid Recovery Support (YORS) NIH HEAL Initiative	Mobile Clinic	Buprenorphine, Naltrexone	Delivered extended-release buprenorphine and extended-release naltrexone via a mobile van that traveled to patient homes
Warrington et al., September, 2020 Vermont, USA	Commercial Laboratory, Aspenti Health	Mobile Clinic	Urine drug testing for OUD patients	Deployed mobile unit to patient homes to conduct urine drug testing on the mobile van
Duncan et al., October, 2020 Minnesota, United States	Hennepin County Jail	Correctional Facility	Buprenorphine	Telehealth-based buprenorphine encounters using a computer in jail medical unit
Roberts et al., September, 2020 New South Whales, Australia	New South Whales Correctional Centers	Correctional Facility	Buprenorphine	Began offering injectable long-acting buprenorphine to people in detention to reduce movement

*OUD* Opioid use disorder; MOUD: Medications for opioid use disorder;* OAT* Opioid agonist therapies,* LAIB* Long acting injectable buprenorphine

#### Expanded telemedicine services

The most common innovation centered on the provision of telemedicine services using telephone or online platforms. Telemedicine services were often instituted to initiate or continue treatment with buprenorphine while minimizing in-person patient-physician encounters. Telemedicine-based buprenorphine was implemented across a range of settings, from nation-wide (Crowley et al. [29] and local hotlines (Samuels et al. [30]) to existing substance use treatment programs (Singh and Tikka [31]; Vecchio et al. [32]; Quiñones et al. [33]), street outreach clinics (Castillo et al. [34]; Tringale et al. [35]; Nordeck et al. [36]; Harris et al. [37]) and correctional facilities (Duncan et al. [38]). Many buprenorphine telemedicine programs also reported prescribing medications for longer durations than usual to reduce the volume of follow up appointments.

Recognizing that many OUD patients do not own mobile phones or have regular internet access to attend buprenorphine telemedicine visits, programs devised creative solutions to ensure receipt of buprenorphine telemedicine services. For instance, Homeless Health Care Los Angeles built sanitized phone booths outside their center to conduct private video calls with their buprenorphine providers (Tringale et al. [35]). In the Pitirre Iniciativa Comunitaria treatment program in Puerto Rico, a private room was set up with a regularly sanitized telephone to allow individual patients to communicate with providers located in a separate room of the same facility (Quiñones et al. [33]). A Boston-based substance use treatment program distributed donated cell phones to facilitate continued telemedicine-based buprenorphine (Komaromy et al. [39]).

In addition to buprenorphine prescribing, telehealth platforms were also used to deliver adjunct psychosocial services to OUD patients. For example, one outpatient MOUD treatment clinic in Rhode Island used telehealth to continue delivering counseling sessions to MOUD patients (Hughto et al. [40]). Another OUD treatment program for pregnant women in Ohio described offering individual and group therapy via telehealth to women in the program to reduce in-person contact (McKiever et al. [41]). In many cases, telehealth and virtual platforms were used as a tool to continue providing outreach and education services throughout the pandemic. For example, one article described how a group of take-home naloxone programs in Ohio shifted their educational and training materials on naloxone use to online platforms (Courser et al. [42]). In a substance use treatment clinic for youth in Massachusetts, text messaging was used to connect and check-in with adolescents and young adults throughout periods of quarantine (Komaromy et al. [39]).

#### Extended take-home medications

Multiple programs in the U.S., Canada, India, Spain, Italy and England instituted longer take-home policies for dispensed opioid agonist medications (Peavy et al. [43], MacKinnon et al. [44], Basu et al. [45], Trujols et al. [46], Vecchio et al. [32] and Hazan et al. [47]). This strategy was facilitated by relaxed regulations and recommendations limiting the prescription of take-home medications, especially around methadone, and a shift in emphasis by regulators on safety and access rather than preventing diversion. These changes were often accompanied by a reduction in requirements for in-person attendance at clinics. One program in India described accommodating vulnerable patients unable to travel to their clinic by dispensing methadone to family members (Ghosh et al. [48]).

#### Uptake of long-acting medications

Another strategy adopted by some programs to reduce the burden of in-person OUD treatment visits was to increase use of long-acting medications (i.e. extended-release naltrexone and buprenorphine). This strategy was adopted by substance use treatment clinics (Straub [49]; Komaromy et al. [39], Wenzel and Fishman [50]), and even a correctional facility in New South Whales, Australia, where long-acting buprenorphine (CAM2038) was made available to help reduce the movement of people throughout the facility (Roberts et al., [51]).

#### Home delivery of services

A few programs described efforts to increase access to services and supplies by delivering them directly to patient homes. In one Maryland-based program, extended-release naltrexone and buprenorphine were delivered directly to patients’ residences and administered outside the home via a mobile van (Wenzel and Fishman [50]). In Ohio, some programs mailed out naloxone kits directly to persons’ residences or arranged for their pick-up at drive-through centers (Courser et al. [42]). Another article described a mobile van service that collected urine drug samples from patients enrolled in OUD treatment by driving directly to their residence (Warrington et al. [52]).

#### Outreach and makeshift services

Multiple programs acknowledged that offering telehealth or home delivery services was a necessary, yet insufficient approach for reaching patients experiencing unstable housing or lacking mobile device ownership. Thus, many programs described the creation of makeshift services or outreach programs to expand the delivery of OUD services during the pandemic. In a Boston-based program (Harris et al. [53]), mobile devices were made available via drop-in centers to facilitate access to buprenorphine visits. One low-threshold buprenorphine program in Baltimore (Nordeck et al. [36]) placed signs on their temporarily closed mobile van with information about how to reach the program by phone, along with a basket of free naloxone kits that was refilled daily. In Tshawne, South Africa, a clinical team distributed methadone through a makeshift health service set up at an emergency homeless shelter (Marcus et al. [54]).

#### Safe supply of opioids

In British Columbia, one program described ‘pandemic withdrawal management’ practices that were implemented in response to provincial Risk Mitigation Guidance that allowed the prescribing of a safe supply of pharmaceutical-grade alternatives to illicit substances, including hydromorphone, stimulants, alcohol, benzodiazepines, and nicotine. Such practices were described to help manage withdrawal symptoms among PWUO by providing a known quality, non-adulterated, pharmaceutical grade alternative to the increasingly toxic illicit drug supply while allowing people to adhere to physical distancing guidelines (MacKinnon et al. [44]). Such practices were adopted in multiple care settings, and were described as especially useful in supportive housing environments that serve PWUOs given easy accessibility and other wraparound services available on site.

### Key obstacles and facilitators for service provision across programs

OUD treatment and harm reduction programs experienced several obstacles throughout the process of adapting their services to COVID-19 circumstances. One commonly reported obstacle included lack of financial resources to support delivery of OUD services. Duncan et al., reported how COVID-19-related costs overwhelmed limited jail healthcare resources, straining the provision of OUD treatment [38]. Marcus et al. [54] described already strained shelter resources that made it extremely difficult to manage hygiene and social distancing among people receiving methadone in a makeshift health service in South Africa. Financial challenges related to the inability of patients to pay for their own medications, such as in the case of patients receiving buprenorphine prescriptions from a syringe exchange program [34]. Others described barriers related to delivering medications to patients, whether due to supply chain issues such as limited interstate transport of medication [45], a lack of pharmacies that stock buprenorphine, as [34], or simply due to the restricted ability of patients and providers to travel long distances during the pandemic [48]. Lastly, other common obstacles included confusion and lack of clear communication by authorities around how to shift MOUD practices (e.g. what patients should be eligible for take home methadone) and COVID social distancing policies (e.g. how to reduce COVID-19 transmission in crowded conditions) [43, 46, 54].

Despite these obstacles, several programs described innovations ensuring continuity of care. First and foremost, most programs acknowledged the critical role of relaxed regulations around MOUD and harm reduction as well as generally greater leniency around OUD service provision that allowed them to provide lower threshold services under COVID-19 conditions [30, 34, 35, 37, 38, 43, 44, 46, 48]. Others acknowledged particular elements of their health system as supporting efforts to provide rapid MOUD services, such as a linked healthcare system in Ireland as described by Crowley et al. [29]. Lastly, some described particular pandemic circumstances that may have additionally facilitated provision of services, such as in the case of a telemedicine program where medical students who were unable to participate in clinical rotations were available to volunteer to run the virtual clinic [34]; or in the case of a Minnesota jail where a lower volume of incarcerated individuals due to COVID-19-motivated jail releases freed up resources to meet demand for MOUD among the jail population [38] (Duncan).

## Discussion

In the current scoping review, we identified innovative policy and program-level efforts that rapidly adapted OUD treatment and harm reduction services to ensure continuous care for PWUOs during the pandemic. Studies covered by this preliminary review were primarily descriptive, highlighting the feasibility of offering telehealth services, increased methadone take-home doses, home delivery of MOUD and naloxone, and prescribing a safe supply of opioid medications for PWUO to reduce risk of overdose from illicit opioids. Strategies to stem disparities in MOUD access for hard-to-reach populations included the provision of long-acting formulations of buprenorphine in place of sublingual buprenorphine in clinics, correctional facilities, and patients’ residences, creation of makeshift services to dispense methadone in emergency shelters, and provision of mobile devices or sanitized phone booths in underserved communities to facilitate engagement with telehealth services. These novel adaptations and experiences of the aforementioned programs under the COVID-19 emergency contribute important practice and policy lessons for the delivery of OUD services that are summarized below:

### Relaxed restrictions on delivery of MOUD

A substantial literature has highlighted the tremendous burden that opioid agonist patients have historically experienced attending frequent in-person clinic visits [55–59]. This burden has been exacerbated and brought to light in the context of COVID-19 risks and restrictions, offering an opportunity to assess alternative models of care [17, 19]. For instance, despite differing views on the risks of overdose and diversion with increased take-home doses of methadone [60] or telemedicine-based opioid treatment with buprenorphine [61], preliminary findings from articles by Nordeck and Hazan suggest rates of treatment retention and mortality did not differ immediately following program adaptations during COVID-19 relative to pre-pandemic outcomes [34, 45]. Furthermore, no studies described diversion or misuse as notable outcomes in adopting more lenient take-home policies or virtual buprenorphine appointments. Indeed, studies prior to the pandemic suggest that increased availability of MOUD may even offset the need for diverting medications for PWUOs experiencing barriers to enrolling in OUD treatment [62].

Despite these promising findings, provider and administrator surveys during the pandemic and prior disasters suggest mixed sentiment to easing restrictions on telemedicine-based visits or extension of methadone take-home doses [60, 63, 64]. In fact, articles by Peavy [43] and Trujols [46] described that some clinics continued to require in-person visits during the pandemic for patients deemed as “high-risk” for overdose or diversion. Hesitancies around take-home doses call for further research to elucidate best practices for balancing risks and benefits of differing take home conditions. These practices also highlight the need for alternative avenues to facilitate ease of access to medication dispensing, such as expanding the role of community pharmacies in dispensing of MOUD [65].

### Remaining challenges in obtaining MOUD

Financial and logistical barriers persisted in some patients’ ability to access medications during the pandemic, as described by Castillo et al. [34]. In cases where long-acting medications were subsidized by grant funding, such as in the study by Wenzel and Fishman, it was unclear how patients would afford long-acting buprenorphine injections following study completion [50]. Issues related to medication cost highlight the need for more prompt changes to funding mechanisms reimbursing MOUD without burdensome co-payments or prior authorization requirements that may disable access [66]. Other unanticipated challenges included limited supply of MOUD due to restrictions on interstate transport during COVID-19 [45]. Such challenges reinforce the need for disaster preparedness guidelines expanding emergency supplies of MOUD in to mitigate disruptions to chronic disease management. With the rising incidence of natural disasters and its disproportionate impact on underserved populations, including PWUOs, well-defined disaster preparedness plans are needed to ensure preparedness and low-threshold access to treatment [67]. The dissemination of disaster preparedness plans and modified treatment guidelines among patients prescribed MOUD, their prescribers, and pharmacists would ensure transparency and a patient-centered approach to mitigate treatment disruption.

Articles described in our review highlight the general safety and feasibility of telemedicine and align with studies prior to the COVID-19 pandemic demonstrating clinical utility among patients receiving treatment for nicotine or alcohol use disorders, [68] or buprenorphine maintenance in rural settings [69]. However, access to telemedicine services is not evenly distributed [70] and portends other challenges to eliminating disparities in OUD treatment. For instance, prior studies among PWUOs have described frequent turnover of mobile phones and phone numbers, lack of access to smartphones, service plans, or internet supporting video visits, inability to secure a private location during telephone encounters, limited staffing and delays to securing FCC Lifeline subsidized phones, and challenges in verifying patient self-report with mandated toxicology testing [68, 71, 72]. Such challenges may be offset by verifying medication adherence with prescription drug monitoring databases, transitioning patients without access to broadband or smartphone technology from video to telephone visits until patients secure subsidized Lifeline phones or internet access, and distributing donated mobile phones as exemplified in programs described by Komaromy et al. [39].

Additional studies are needed to identify systems-, provider-, and patient-level factors influencing the delivery of telehealth services, and exploring which patient sub-groups would benefit from telephone-, video-, and/or in-person encounters with their providers. This includes better understanding and providing guidance around which patients are at risk of harm from relaxed medication restrictions, if any, and whether and how such risks should inform decisions around appropriate use of telehealth or take-home schedules for medications such as methadone. In addition, more work is needed to assess the role of integrated care models in easing patient access to specialty care (e.g., psychiatry, infectious diseases) or social services during emergencies.

### Implications for expanding harm reduction efforts

Finally, reviewed articles contributed some important insights around the delivery of harm reduction services during the pandemic. Mailing or coordinating in-person pick-up of Naloxone as described by Nordeck [36] and Courser [42] highlight patient-centered approaches that could complement efforts to facilitate access to naloxone, such as efforts to offer naloxone as an over-the-counter medication [73]. These distribution strategies can be combined with online modules reinforcing opioid overdose education [74]. Such online training modules may be standardized, delivered in a synchronized or asynchronized format, and disseminated broadly to increase the availability of naloxone and overcome stigma towards PWUOs in communities at highest risk [75].

The provision of safe supply of opioids in British Columbia for PWUOs described by MacKinnon and colleagues [44] resulted in high provider and patient satisfaction[44], emphasizing the need to further consider such harm reduction approaches that mitigate patient exposure to an increasingly lethal illicit opioid supply [76, 77]. Longer-term studies are underway to assess the role of a prescribed supply of opioids as alternatives to illicit opioids [22], and more is needed to understand ideal duration for prescriptions, their role in stabilizing patients, best practices for dissemination and how to address emerging political-legal concerns.

### Limitations

Our review is subject to multiple limitations, including being confined to studies published in English in 2020, those describing specific OUD treatment or harm reduction program modifications during the pandemic, and excluding the grey literature. Thus, it is likely that many innovations from non-English speaking or non-academic settings, or which were published in 2021 were not assessed. Furthermore, programs described in this review often lacked long-term health outcomes and require subsequent prospective and retrospective analyses to clarify their safety and clinical impact over time. While most articles claimed program adaptations were generally well-received by OUD patients and staff, robust and longer-term studies are needed to rigorously identify programmatic and policy modifications that optimize uptake of MOUD and harm reduction services and improve patient outcomes.

## Conclusion

The COVID-19 pandemic has posed unprecedented challenges globally for PWUOs. Nevertheless, it has also accelerated the development and scalability of innovations and transformed policies, care models, and technologies to lower thresholds for life-saving treatment and harm reduction services. The pandemic has further confirmed the need for additional studies, revision of ethical and legal frameworks, and additional patient- and provider-driven innovations around the provision of OUD services. As we continue to work to confine the ongoing overdose and COVID-19 crises, we hope that lessons learned from the rapid innovations in OUD services will bring lasting changes and improvements in the delivery of effective and humane care.

## Data Availability

Data sharing is not applicable to this article as no datasets were generated or analyzed during the current study.

## Appendix 1

See Table 3.

**Table 3:** PRISMA Checklist, adapted from Tricco et al. [24]

Section	Item	PRISMA-ScR checklist item	Status
Title			
Title	1	Identify the report as a scoping review	Done
Abstract			
Structured summary	2	Provide a structured summary that includes (as applicable): background, objectives, eligibility criteria, sources of evidence, charting methods, results, and conclusions that relate to the review questions and objectives	Done
Introduction			
Rationale	3	Describe the rationale for the review in the context of what is already known. Explain why the review questions/objectives lend themselves to a scoping review approach	Done
Objectives	4	Provide an explicit statement of the questions and objectives being addressed with reference to their key elements (e.g., population or participants, concepts, and context) or other relevant key elements used to conceptualize the review questions and/or objectives	Done
Methods			
Protocol and registration	5	Indicate whether a review protocol exists; state if and where it can be accessed (e.g., a Web address); and if available, provide registration information, including the registration number	NA
Eligibility criteria	6	Specify characteristics of the sources of evidence used as eligibility criteria (e.g., years considered, language, and publication status), and provide a rationale	Done
Information sources*	7	Describe all information sources in the search (e.g., databases with dates of coverage and contact with authors to identify additional sources), as well as the date the most recent search was executed	Done
Search	8	Present the full electronic search strategy for at least 1 database, including any limits used, such that it could be repeated	Done
Selection of sources of evidence†	9	State the process for selecting sources of evidence (i.e., screening and eligibility) included in the scoping review	Done
Data charting process‡	10	Describe the methods of charting data from the included sources of evidence (e.g., calibrated forms or forms that have been tested by the team before their use, and whether data charting was done independently or in duplicate) and any processes for obtaining and confirming data from investigators	Done
Data items	11	List and define all variables for which data were sought and any assumptions and simplifications made	Done
Critical appraisal of individual sources of evidence§	12	If done, provide a rationale for conducting a critical appraisal of included sources of evidence; describe the methods used and how this information was used in any data synthesis (if appropriate)	NA
Synthesis of results	13	Describe the methods of handling and summarizing the data that were charted	Done
Results			
Selection of sources of evidence	14	Give numbers of sources of evidence screened, assessed for eligibility, and included in the review, with reasons for exclusions at each stage, ideally using a flow diagram	Done
Characteristics of sources of evidence	15	For each source of evidence, present characteristics for which data were charted and provide the citations	Done
Critical appraisal within sources of evidence	16	If done, present data on critical appraisal of included sources of evidence (see item 12)	NA
Results of individual sources of evidence	17	For each included source of evidence, present the relevant data that were charted that relate to the review questions and objectives	Done
Synthesis of results	18	Summarize and/or present the charting results as they relate to the review questions and objectives	Done
Discussion			
Summary of evidence	19	Summarize the main results (including an overview of concepts, themes, and types of evidence available), link to the review questions and objectives, and consider the relevance to key groups	Done
Limitations	20	Discuss the limitations of the scoping review process	Done
Conclusions	21	Provide a general interpretation of the results with respect to the review questions and objectives, as well as potential implications and/or next steps	Done
Funding			
Funding	22	Describe sources of funding for the included sources of evidence, as well as sources of funding for the scoping review. Describe the role of the funders of the scoping review	Done

## Abbreviations

PWUO: People who use opioids
OUD: Opioid use disorder
MOUD: Medications for opioid use disorder
COVID-19: Novel Coronavirus disease 2019
